# Does dose calculation algorithm affect the dosimetric accuracy of synthetic CT for MR‐only radiotherapy planning in brain tumors?

**DOI:** 10.1002/acm2.70030

**Published:** 2025-02-19

**Authors:** Jeffrey C. F. Lui, Francis K. H. Lee, C. C. Law, James C. H. Chow

**Affiliations:** ^1^ Department of Clinical Oncology Queen Elizabeth Hospital Hong Kong China

**Keywords:** AcurosXB, analytical anisotropic algorithm, brain, deep‐learning, MR‐only, synthetic CT

## Abstract

**Purpose:**

This study compares the dosimetric accuracy of deep‐learning‐based MR synthetic CT (sCT) in brain radiotherapy between the Analytical Anisotropic Algorithm (AAA) and AcurosXB (AXB). Additionally, it proposes a novel metric to predict the dosimetric accuracy of sCT for individual post‐surgical brain cases.

**Materials and Methods:**

A retrospective analysis was conducted on 20 post‐surgical brain tumor patients treated with Volumetric Modulated Arc Therapy (VMAT). sCT and planning CT images were obtained for each patient. Treatment plans were optimized on sCT and recalculated on planning CT using both AAA and AXB. Dosimetric parameters and 3D global gamma analysis between sCT and planning CT were recorded. The bone volume ratio, a novel metric, was calculated for each patient and tested its correlation with gamma passing rates.

**Results:**

For AAA, the mean differences in D_mean_ and D_max_ of PTV between sCT and planning CT were 0.2% and −0.2%, respectively, with no significant difference in PTV (*p* > 0.05). For AXB, mean differences in D_mean_ and D_max_ of PTV were 0.3% and 0.2%, respectively, with significant differences in D_mean_ (*p* = 0.016). Mean gamma passing rates for AXB were generally lower than AAA, with the most significant drop being 9.3% using 1%/1 mm analyzed in PTV. The bone volume ratio showed significant correlation with gamma passing rates.

**Conclusions:**

Compared to AAA, AXB reveals larger dosimetric differences between sCT and planning CT in brain photon radiotherapy. For future dosimetric evaluation of sCT, it is recommended to employ AXB or Monte Carlo algorithms to achieve a more accurate assessment of sCT performance. The bone volume ratio can be used as an indicator to predict the suitability of sCT on a case‐by‐case basis.

## 1 INTRODUCTION

The need for MR synthetic CT (sCT) in radiotherapy arises from the increasing utilization of MRI in radiotherapy treatment planning. While MRI provides superior soft tissue contrast and is valuable for target delineation, it does not generate electron density information for accurate dose calculations. Traditionally, planning CT has been the standard to provide electron density data, but the overall treatment planning process will suffer registration errors between CT and MRI.^1^ The use of sCT solved the dilemma by converting MR images into CT‐like images. This enables seamless integration of MRI into the radiotherapy workflow, improves treatment planning accuracy,^2^ reduces patient inconvenience from multiple imaging sessions,^3^ and provides possibilities for adaptive radiotherapy with real‐time MRI guidance.^4^, ^5^, ^6^

Brain is one of the regions that can benefit from the use of sCT due to the crucial role of MRI in brain tumor delineation.^7^, ^8^ Amongst the numerous algorithms proposed for generating sCT in the brain,^9^, ^10^, ^11^, ^12^, ^13^, ^14^, ^15^ a common limitation is the inaccurate tissue labeling at the bone/air interface,^9^, ^16^, ^17^, ^18^, ^19^ such as in regions near the sinus cavity and auditory canal. This limitation arises primarily from the similarity in signal intensities between air and bone in MRI. Although the use of dedicated sequences (e.g., Ultra‐short Echo Time [UTE] sequence)^9^ or deep‐learning‐based algorithms^12^, ^15^ has improved the differentiation between air and bone, this problem persists due to partial volume effect, artifact, noise and image misalignment.^9^, ^20^

Another factor that can cause tissue mislabeling in sCT is the presence of anatomical anomalies, such as missing tissue or metal implants after brain surgery. Literature showed significant CT number discrepancies between planning CT and sCT generated by atlas‐based algorithm for patients with partial skull removal during surgery.^10^ In these cases, the sCT incorrectly generated a segment of bone in the area where the bone was missing. Although the adoption of deep‐learning‐based algorithms in sCT reconstruction has shown promising improvements in handling missing tissue,^12^, ^21^ the reconstruction is not flawless, as noticeable CT number differences were still observed in the vicinity of anatomical anomalies.^21^

Many studies have evaluated the dosimetric accuracy of sCT for the brain region by comparing the dose distribution calculated on sCT with that on planning CT.^19^, ^21^, ^22^, ^23^ Although the results have consistently demonstrated that the dosimetric discrepancies between sCT and planning CT were insignificant even in the presence of tissue mislabeling, most of the evaluations have predominantly relied on convolution‐and‐superposition‐based algorithms, such as the Analytical Anisotropic Algorithm (AAA) or Collapsed Cone Calculation (CCC) algorithm, for dose calculation. These algorithms calculate and report dose‐to‐water, which diverges from recent guidelines and literature,^24^, ^25^ recommending the use of algorithms on dose‐to‐medium for a more coherent dose evaluation. Furthermore, studies have indicated that convolution‐and‐superposition‐based algorithms may have sub‐optimal accuracy in the presence of tissue heterogeneity,^26^, ^27^, ^28^, ^29^ such as the bone/air interface in sinus cavities and bone/soft tissue interfaces in the skull. Therefore, it is preferable to use more sophisticated algorithms, including deterministic radiation transport or Monte Carlo, which can better account for tissue heterogeneity^27^, ^28^, ^29^, ^30^, ^31^ in dosimetric evaluation.

The objective of this study is to compare the dosimetric accuracy of the sCT generated by a commercially available deep‐learning‐based sCT software in brain radiotherapy, between convolution‐and‐superposition‐based dose algorithm (AAA) and deterministic radiation transport dose algorithm (AcurosXB). Additionally, we aimed to propose an sCT‐based metric to predict the dosimetric accuracy of sCT for each individual post‐surgical brain case, thus assisting to determine the applicability of sCT in MR‐only treatment planning.

## 2 MATERIALS AND METHODS

### 2.1 Patient data and image acquisition

This study was approved by the Research Ethics Committee of the Hospital. A total of 20 patients with brain tumors were retrospectively selected from those who received volumetric modulated arc therapy (VMAT) at our hospital, between June 2022 and December 2023 (aged 29 to 75 in 2023, 9 patients with glioma and 11 patients with meningioma). The prescription dose to the Planned Target Volume (PTV) ranged from 40 to 60 Gy, delivered in 10 to 33 fractions. The mean PTV volume was 224.7 cm^3^, ranging from 41.0 to 475.8 cm^3^. All patients in this study have undergone preceding brain surgery, in which one of the patients received a piece of custom‐shaped synthetic material as substitute for the skull at the surgical opening.

For each patient, both sCT and planning CT scans were acquired. To generate the sCT, T1 VIBE DIXON image (1.6 mm × 1.6 mm × 1.5 mm) was obtained using a 1.5 T MRI (MAGNETOM Aera, Siemens Healthcare GmbH, Germany) equipped with a flat couch top and an MRI‐compatible external laser system (DORADOnova MR3T, LAP GmbH Laser Applikationen, Germany). The imaging parameters of the sequence were provided by the vendor (bandwidth = 96 kHz, repetition time = 6.7 ms, echo time = 2.4 ms). The mean image acquisition time is 3.7 min. Patient immobilization was achieved using the Type‐S Overlay board (CIVCO, Iowa, USA), along with thermoplastic masks, provil spacer, and head‐and‐neck Vac‐Lok cushions. Patient positioning was performed by aligning the preliminary isocenter marked by the oncologist on the thermoplastic mask with the laser iso‐center. An 18‐channel body flex coil was utilized to cover the scan region, with a coil bridge to support its weight and secure its position relative to the thermoplastic mask. Additionally, a posterior spine coil was used to ensure sufficient signal reception at the posterior of the brain.

The T1 VIBE DIXON image was processed by a deep‐learning‐based sCT reconstruction software (VB60, Syngo.via RT Image Suite, Siemens Healthcare GmbH, Germany) to generate the sCT (1.0 mm × 1.0 mm × 1.5 mm). This software utilizes two networks: (1) a Convolutional Neural Network is employed to segment the input MR image into background, bone, and soft tissue. (2) these segmentations and the input MR image are passed into a Generator and Discriminator Network to reconstruct an sCT with continuous HU values.

Planning CT images (1.2 mm × 1.2 mm × 3 mm) were obtained in a CT simulator (Brilliance 16 Big Bore, Koninklijke Philips N.V., the Netherlands). Patients were positioned in the treatment posture using the same immobilization device and imaging iso‐center as in the MR simulation. The time between MR and CT imaging sessions was 5 days on average.

### 2.2 Image agreement evaluation and dosimetric evaluation

To compare the CT number between sCT and planning CT, pixel‐to‐pixel CT number mean absolute error (MAE) and mean error (ME) were calculated for overall body, brain, and skull bone regions for all patients. In addition, the Dice similarity coefficient (DSC) was calculated to compare the skull bone segmentation between sCT and planning CT. The skull bone segmentation was identified by thresholding the tissue at 100 HU or above on skull region.

For dosimetric evaluation, VMAT treatment plans were generated for all 20 patients using 6 MV photon beams. These plans were optimized based on the sCT using treatment planning system (Eclipse 16.1, Varian Medical Systems, USA). The target dose was based on the oncologist's prescription, and dose constraints followed published guidelines.^32^, ^33^, ^34^ The optimized VMAT plans were transferred to the planning CT for dose calculation. The same grid size of 2.5 mm and CT‐relative electron density calibration curve were applied for dose calculation on both the sCT and planning CT. The dose was calculated using both AAA and AcurosXB (AXB) with the 13.5 physical material table in Eclipse, and the results were reported as dose‐to‐water for AAA and dose‐to‐medium for AXB. Since the immobilization devices were not visible in the sCT, their attenuation was excluded in the dose calculation volume of planning CT for a fair comparison.

Clinically relevant dose metrics between the sCT and planning CT were compared for AAA and AXB calculated plans. For PTV, metrics included the D_max_, D_mean_, D_95_, V_95_, heterogeneity index (HI), and RTOG conformity index (CI). HI is defined as (D_2_‐D_98_)/D_50_ × 100. A lower CI value indicate better dose homogeneity. CI is defined as V_RI_/TV, where V_RI_ is the prescription isodose volume and TV is the PTV volume. A CI value closer to 1 indicates better dose conformity to the PTV. For OARs, the metrics included D_max_ for serial organs (brainstem, optic chiasm, optic nerves, and lens) and D_mean_ for parallel organs (cochlea). Wilcoxon sign‐ranked test was performed on these comparisons. Additionally, 3D global gamma analysis was conducted. To achieve a comprehensive evaluation, the gamma analysis was performed in the entire imaged volume and repeated in the PTV. Each of these analyses was performed using the dose difference and distance‐to‐agreement (DTA) criteria of 3%/2, 3%/1, 2%/1, and 1%/1 mm, with 10% low‐dose‐threshold.

Moreover, a novel sCT‐based metric, bone volume ratio, was calculated for each patient. This metric is defined as the ratio of bone volume within PTV to the volume of PTV, in which the bone was identified as the tissue with 100 HU or above in the sCT. Pearson correlation test was performed to assess the correlation between the bone volume ratio and the gamma passing rates.

## 3 RESULTS

### 3.1 Image agreement evaluation and dosimetric evaluation

The mean CT number MAE and ME (± 1 standard deviation) between sCT and planning CT among the 20 patients were 112.8 ± 13.7 HU and −14.6 ± 25.7 HU for the overall body, 13.4 ± 1.6 HU and −8.9 ± 2.1 HU for the brain, and 236.8 ± 20.0 HU and 4.0 ± 62.9 HU for the skull bone. The mean DSC for the skull bone among the 20 patients was 0.87 ± 0.02. These results are consistent with findings from previous literature^19^ that evaluated the same sCT reconstruction algorithm used in this study.

**FIGURE 1:**
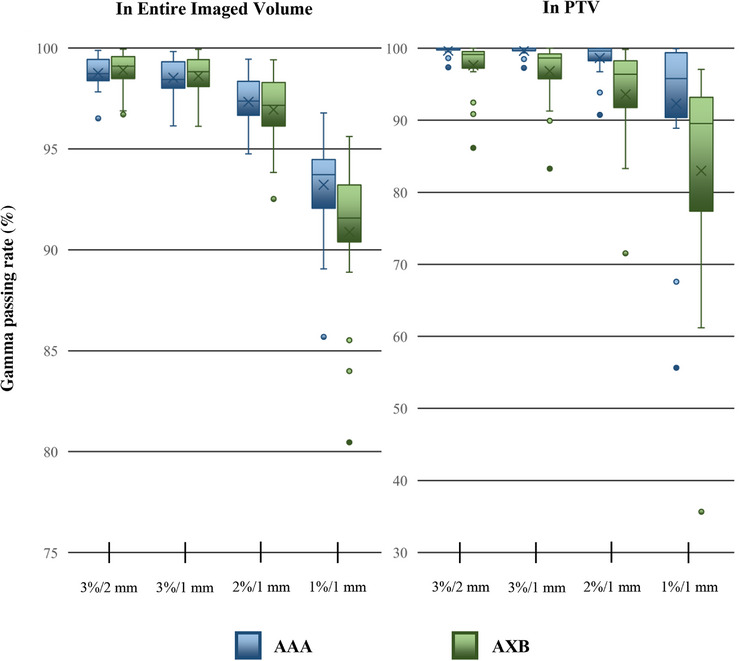
The gamma passing rates between the dose distribution calculated in planning CT and sCT using AAA and AXB. AAA, Analytical Anisotropic Algorithm; AXB, AcurosXB; sCT, synthetic CT.

The mean PTV dosimetric differences among 20 patients were presented in Table 1. For AAA calculated treatment plans, the mean differences between planning CT and sCT for PTV dose metrics were: D_max_ (−0.2%), D_mean_ (0.2%), D_95_ (0.1%), and V_95_ (0.0%). No statistically significant differences were found in any PTV dose metrics. For OAR dose metrics, brainstem D_max_ (1 out of 8 structures) showed a statistically significant difference between sCT and planning CT (*p* = 0.047). The mean gamma passing rate for AAA exceeded 90% in all analysis settings, as depicted in Figure 1. This shows that with the use of AAA, there is little dosimetric difference between those calculated on sCT and planning CT.

**TABLE 1:** The mean dose metrics differences (planning CT minus sCT) of 20 patients.

	AAA		AXB	
	Mean Difference (Range)	*p*‐Value	Mean Difference (Range)	*p*‐Value
PTV D_max_ (%)	−0.2 (−2.5 to 0.8)	0.768	0.2 (−3.4 to 1.4)	0.231
PTV D_mean_ (%)	0.2 (−0.3 to 1.0)	0.076	0.3 (−0.4 to 1.8)	**0.016**
PTV D_95_ (%)	0.1 (−0.4 to 0.9)	0.312	0.0 (−2.0 to 2.0)	0.784
PTV V_95_ (%)	0.0 (−0.1 to 0.2)	0.359	0.0 (−0.4 to 0.6)	0.698
HI	−0.1 (−1.7 to 0.0)	0.243	0.0 (−0.0 to 0.0)	0.121
CI	0.0 (−0.0 to 0.0)	0.881	0.0 (−0.2 to 0.1)	0.352
Brainstem D_max_ (cGy)	10.7 (−5.7 to 47.4)	**0.047**	12.8 (−31.8 to 100.2)	**0.003**
Optic chiasm D_max_ (cGy)	−7.5 (−67.8 to 14.5)	0.687	10.7 (−31.8 to 100.2)	0.105
L Len D_max_ (cGy)	5.6 (−5.3 to 124.1)	0.191	5.7 (−4.3 to 142.7)	**0.044**
R Len D_max_ (cGy)	5.3 (−7.6 to 12.9)	0.695	0.0 (−28.0 to 25.4)	0.870
L Optic nerve D_max_ (cGy)	−3.1 (−74.9 to 21.7)	0.984	14.8 (−20.2 to 162.5)	0.107
R Optic nerve D_max_ (cGy)	−4.6 (−80.6 to 31.5)	0.571	16.8 (−22.2 to 60.6)	**0.003**
L Cochlea D_mean_ (cGy)	12.3 (−6.3 to 83.2)	0.073	9.4 (−11.3 to 66.6)	0.167
R Cochlea D_mean_ (cGy)	38.8 (−15.4 to 115.1)	0.154	−7.7 (−84.3 to 27.2)	**0.022**

*Note*: Bold *p‐value = *< 0.05 is considered statistically significant.

Abbreviations: AAA, Analytical Anisotropic Algorithm; AXB, AcurosXB; CI, RTOG conformity index; HI, heterogeneity index; L, left; PTV, planning target volume; R, right.

For AXB calculated treatment plans, the mean differences between planning CT and sCT for PTV dose metrics were: D_max_ (0.2%), D_mean_ (0.3%), D_95_ (0.0%), and V_95_ (0.0%). Statistically significant differences were found in D_mean_ of PTV (*p* = 0.016). For OAR dose metrics, 4 out of 8 structures showed statistically significant difference between sCT and planning CT, including brainstem D_max_ (*p* = 0.003), left len D_max_ (*p* = 0.044), right optic nerve D_max_ (*p* = 0.003), and right cochlea D_mean_ (*p* = 0.022). As depicted in Figure 1, the mean gamma passing rates for AXB are generally lower than that of AAA. For instance, the mean 1%/1 mm gamma passing rate analyzed in entire imaged volume and PTV decreased to 90.9% and 83.0% respectively for AXB.

### 3.2 Bone volume ratio

**FIGURE 2:**
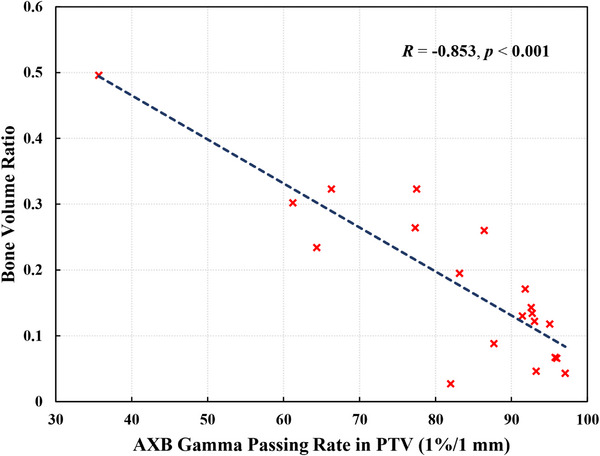
Scatter plot between the bone volume ratio and the 1%/1 mm gamma passing rate in PTV with 10% low‐dose‐threshold for AXB. AXB, AcurosXB; PTV, planning target volume.

The bone volume ratio of the 20 patients ranged from 0.027 to 0.496, with an average of 0.184 and a standard deviation of 0.121. The results of the Pearson correlation tests were listed in Table 2, which indicated moderate to strong negative correlations between the bone volume ratio and the gamma passing rates for AXB. The scatter plot in Figure 2 demonstrates the strong correlation between the bone volume ratio and the 1%/1 mm gamma passing rate analyzed in the PTV for AXB (*R* = −0.853, *p* < 0.001).

**TABLE 2:** Pearson correlation coefficient between Gamma passing rates and the bone volume ratio.

	AAA		AXB	
	*R*	*p*‐Value	*R*	*p*‐Value
	**In entire imaged volume**			
**3% / 2** **mm**	−0.134	0.575	−0.440	0.052
**3% / 1** **mm**	−0.112	0.639	−0.450	**0.047**
**2% / 1** **mm**	−0.170	0.474	−0.546	**0.013**
**1% / 1** **mm**	−0.281	0.230	−0.607	**0.005**
	**In PTV**			
**3% / 2** **mm**	−0.532	**0.016**	−0.498	**0.026**
**3% / 1** **mm**	−0.582	**0.007**	−0.517	**0.020**
**2% / 1** **mm**	−0.756	**<0.001**	−0.725	**<0.001**
**1% / 1** **mm**	−0.658	**0.002**	−0.853	**<0.001**

*Note*: Bold *p‐value = *< 0.05 is considered as statistically significant. Gamma analysis were performed with 10% low‐dose‐threshold.

Abbreviations: AAA, Analytical Anisotropic Algorithm; AXB, AcurosXB.

## 4 DISCUSSION

The brain is one of the most frequently targeted regions for MR‐only treatment planning. Due to its unique challenges for sCT, including the presence of air cavities and potential anatomical anomalies from brain surgery, identifying the most accurate dosimetric evaluation methods for assessing sCT in this region is essential. To the best of our knowledge, this article represents the first attempt to compare the dosimetric accuracy of a deep‐learning‐based sCT algorithm for brain photon radiotherapy using different types of dose calculation algorithms, including convolution and superposition methods (AAA), and the deterministic radiation transport method (AXB).

Earlier studies have sought to evaluate the impact of dose algorithms on sCT dosimetric accuracy. Kazemifar et al.^35^ assessed an in‐house developed deep‐learning‐based sCT algorithm for brain proton therapy using pencil beam and Monte Carlo algorithms. Their study found comparable dose differences between sCT and planning CT for both algorithms. However, given the significant differences in dose deposition mechanisms between protons and photons, it is essential to evaluate the effects of dose calculation algorithms on sCT dosimetric accuracy specifically in photon radiotherapy. Additionally, Qi et al.^36^ and Prunaretty et al.^37^ examined the dosimetric accuracy of sCT in photon therapy using various dose algorithms, both noting that the dosimetric differences between sCT and planning CT were greater with advanced algorithms such as AXB or Monte Carlo. Nevertheless, their investigations were confined to the head and neck, as well as the pelvis regions, leaving the impact on the brain region unresolved.

Many existing evaluations of sCT algorithms on brain photon radiotherapy have relied on convolution and superposition methods for dosimetric assessment^11^, ^14^, ^18^, ^21^, ^22^ or have not specified the type of dose algorithm employed.^13^, ^19^ Given the limitations of the current sCT algorithms, in particular reconstructing bone at the interface with air or soft tissue in brain, it is believed that more sophisticated dose algorithms will result in more dosimetric discrepancy when sCT is used. Deterministic radiation transport and Monte Carlo algorithm are known for their superior dosimetric accuracy and are widely adopted. This problem in sCT treatment planning for brain region cannot be overlooked.

Our study confirmed that the dosimetric accuracy of sCT worsened when AXB is used. Specifically, when AAA was employed to compare sCT and planning CT, no statistically significant dosimetric differences were found in PTV, and only one out of eight OAR dose metrics showed statistically significant difference. Additionally, the mean gamma passing rates exceeded 90% for all analysis. This result is consistent with previous dosimetric studies for deep‐learning‐based sCT algorithm utilizing AAA.^14^, ^21^, ^22^ However, when using the AXB algorithm, statistically significant differences were found in PTV D_mean_ and four out of eight OAR dose metrics. The mean gamma passing rates also decreased across most analysis settings. For instance, the mean gamma passing rate in PTV decreased by 2.0% (3%/2 mm), 2.8% (3%/1 mm), 5.1% (2%/1 mm) and 9.3% (1%/ 1  mm). In the most extreme case, the gamma passing rate in PTV decreased by 12.6% (3%/2 mm), 15.2% (3%/1 mm), 19.2% (2%/1 mm) and 20.0% (1%/ 1  mm).

**FIGURE 3:**
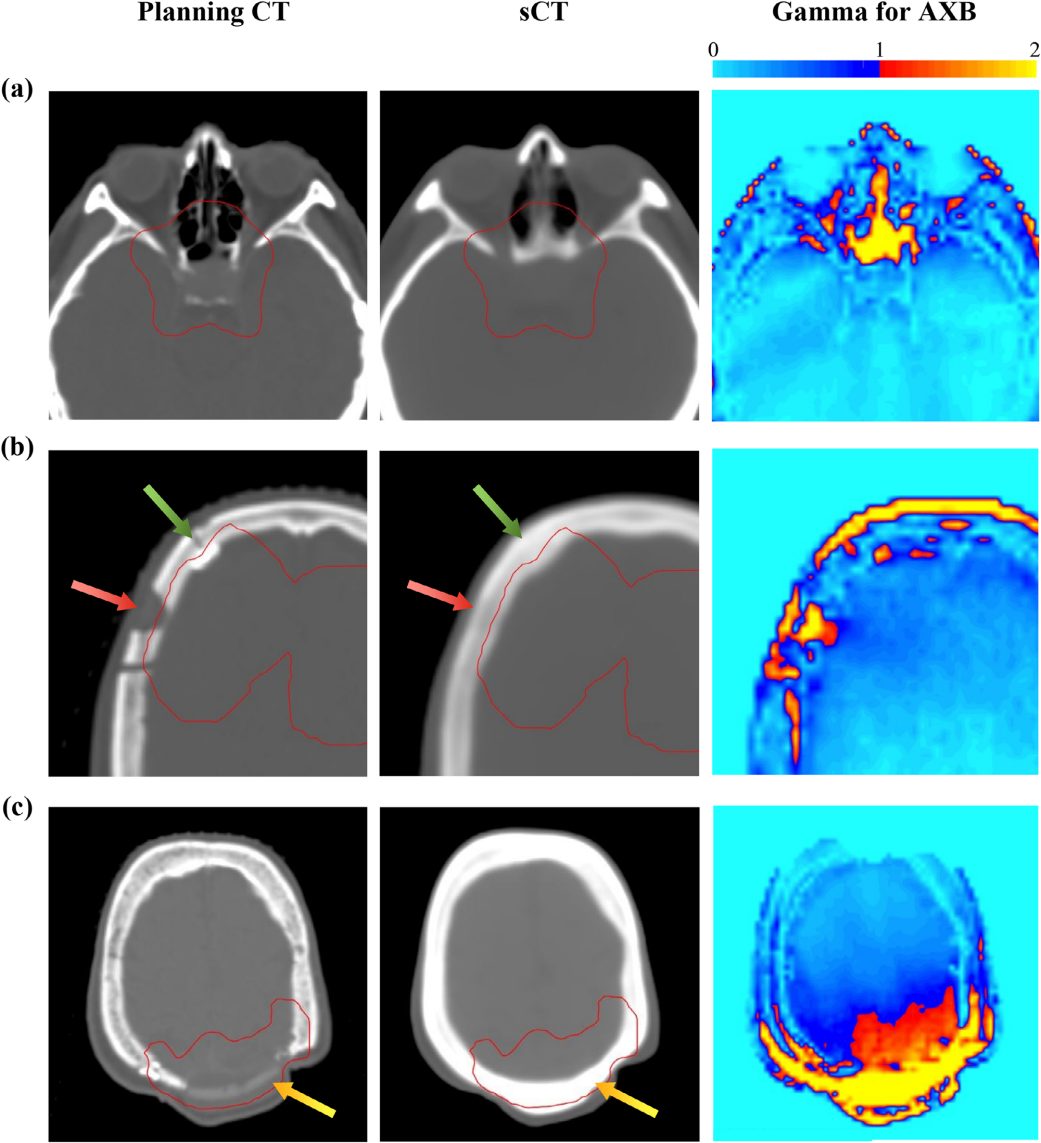
The planning CT (left), sCT (middle), and the 1%/1 mm global gamma distribution in entire imaged volume with a 10% low‐dose‐threshold for AXB (right) of three sample patients. The red contour in the planning CT and sCT outlines the PTV. (a) presents a case where the PTV is located near the sinus cavities. (b) presents a case with a post‐surgery drill hole and a metal screw (indicated by the red and green arrows, respectively), which were mislabeled as bone in the sCT. (c) presents a case with the use of synthetic material for skull contour restoration (indicated by the yellow arrow), which was mislabeled as bone in the sCT. PTV, planning target volume; sCT, synthetic CT.

This significant discrepancy can be attributed to the different nature of dose calculation and reporting in the AAA and AXB algorithms. AAA inherently calculates and reports dose‐to‐water, which means that for the same incident fluence, the dose deposited in any material is considered identical, as all materials are treated as water in terms of their chemical composition. On the other hand, AXB calculates and reports dose‐to‐medium intrinsically, leading to noticeable differences between the dose deposited in bone and that in soft tissue or air due to their distinct mass stopping power ratios.^38^ According to literature, this difference can be as high as 11% for cortical bone.^39^ While this difference is generally small in clinical situations as actual bone is less dense than cortical bone,^25^ it explains the reduced dosimetric accuracy of sCT with AXB, especially at the edge of skull and sinus cavities, where tissue mislabelling is most commonly encountered (Figure 3a). Such discrepancy is also expected in the Monte Carlo algorithm, as it shares similarities with the AXB algorithm in terms of the dose‐to‐medium nature and accuracy in handling tissue heterogeneity.^27^, ^30^ Given its systematic nature and impact on most patients, this dosimetric inaccuracy of sCT should not be overlooked when deciding whether to adopt sCT over the planning CT in treatment planning.

All patients included in this study have undergone prior brain surgery. The anatomical anomalies resulting from the surgery were expected to pose additional challenges in sCT reconstruction. This study revealed that the drill holes and metal screws used to secure the reinserted bone after brain surgery were frequently mislabeled as bone in sCT. This mislabeling could perturb the dosimetric accuracy in the surrounding region, as demonstrated in Figure 3b where noticeable failures in gamma analysis occur at or near the drill holes and metal screws. Hence, to improve dosimetric accuracy, it is recommended to apply manual CT number overrides to the mislabeled drill holes and metal screws that are clearly visible on MR images.

This study introduced a novel metric, the bone volume ratio, to quantify the proportion of bone within the target volume. The motivation behind this metric arose from the observation that large dose deviations occurred near the bone interfaces, such as near sinus cavities and surgical‐induced anomalies on the skull. We hypothesized that the bone volume ratio could effectively predict the dosimetric accuracy of sCT. Our results validated this hypothesis by demonstrating significant negative correlations between the bone volume ratio and the gamma passing rates of the AXB. The bone volume ratio can be utilized in the MR‐only treatment planning workflow as an indicator to identify cases with potential significant dosimetric inaccuracies in sCT. These cases can then be directed to the traditional treatment planning workflow with planning CT for more reliable dose calculations.

It is worth noting that, due to the lack of consensus on clinically acceptable gamma passing rates for sCT evaluation, we do not propose a specific cut‐off value for the bone volume ratio in this paper. Furthermore, as this study included only post‐surgical patients, further validation is needed to evaluate the generalizability of bone volume ratio in non‐surgical cases. Therefore, institutions are recommended to validate the prediction power of bone volume ratio on their clinical cases and establish cut‐off values based on their desired level of gamma passing rate.

There is an outlier patient in this study who underwent skull contour restoration using a custom‐shaped synthetic material (Figure 3c). Despite the synthetic material having a lower physical density than the skull bone, it was incorrectly reconstructed as bone in the sCT due to their similar level of MR signal. Consequently, substantial dose discrepancies were observed in the adjacent PTV, leading to the largest dose difference between sCT and planning CT (1.8% for PTV's D_mean_ for AXB) and the lowest gamma passing rates (35.7% for 1%/1 mm gamma passing rate analyzed in PTV for AXB) among all patients. This example highlights the importance of carefully evaluating the material of surgical implants and their reconstruction in sCT when assessing the suitability of sCT treatment planning.

## 5 CONCLUSIONS

Compared to AAA, using AXB for dose calculation reveals greater dosimetric discrepancies between sCT and planning CT in brain photon radiotherapy. For future dosimetric evaluations of sCT, it is recommended to utilize AXB or Monte Carlo algorithms for a more accurate assessment of sCT performance. In clinical applications, the suitability of sCT should be evaluated on a case‐by‐case basis. One potential approach is to incorporate the bone volume ratio as an indicator to predict the dosimetric accuracy of sCT for each individual case.

## AUTHOR CONTRIBUTIONS

**Jeffrey C. F. Lui**: Conceptualization; data curation; formal analysis; investigation; methodology; project administration; supervision; validation; visualization; writing—original draft; writing—review & editing. **Francis K. H. Lee**: Conceptualization; methodology; project administration; supervision; validation; writing—original draft; writing—review & editing. **C. C. Law**: Conceptualization; validation; writing—review & editing. **James C. H. Chow**: Conceptualization; validation; writing—review & editing.

## CONFLICT OF INTEREST STATEMENT

The authors declare no conflict of interest.
